# *In Vitro* and* In Vivo* Biofilm Characterization of Methicillin-Resistant *Staphylococcus aureus* from Patients Associated with Pharyngitis Infection

**DOI:** 10.1155/2016/1289157

**Published:** 2016-09-28

**Authors:** Shanmugaraj Gowrishankar, Arumugam Kamaladevi, Krishnaswamy Balamurugan, Shunmugiah Karutha Pandian

**Affiliations:** Department of Biotechnology, Alagappa University, Science Campus, Karaikudi, Tamil Nadu 630 003, India

## Abstract

The present investigation was deliberately aimed at evaluating the biofilm-forming ability of 63 clinical MRSA isolates recovered from pharyngitis patients through different phenotypic assays. The molecular detection of adhesion (*icaA/icaD/icaB/icaC*), adhesins (*fnbA/fnbB*,* clfA*, and* cna*), staphylococcal accessory regulator (*sarA*), and *α*-toxin (*hla*) genes was done by employing polymerase chain reaction (PCR). Out of 63 isolates, 49 (77.8%) were found slime positive by the Congo red agar (CRA) method and 44 (69.8%) as biofilm positive by the quantitative microtitre plate assays. The results of MATH assay showed that most of the test pathogens are hydrophilic in nature. The molecular investigation of biofilm-associated genes revealed that 84.13% (*n* = 53) of isolates were found positive for* icaADBC* genes. The* fnbA* and* fnbB* genes were present in 49 (77.8%) and 51 (81%) MRSA isolates, respectively. In addition, 58.7% (*n* = 37), 73% (*n* = 46), and 69.8% (*n* = 44) of the isolates harboured the* clfA*,* cna*, and* hla* genes, respectively. Further, nearly 81% (*n* = 51) of the isolates were found positive for the gene* sarA* and all the* ica* negative isolates were also negative for the gene. Furthermore, the results of* in vivo* adherence assay unveiled the factual commonness in the* in vitro* adherence method.

## 1. Introduction

Globally, myriad of bacterial pathogens inhabiting the environment cause several acute and chronic infections to human through their ability to form dynamic, structurally complex, and multilayered cellular matrix, termed as biofilms [1]. The synthesis of such biofilms by pathogenic bacteria is therefore considered to be a major virulence factor, since the recalcitrant biofilms comprehensively safeguard the pathogens not only from host defence mechanism but also from the targeted action of therapeutic drugs [2]. Methicillin-resistant* Staphylococcus aureus* (MRSA) continues to be the most prominent biofilm-forming human pathogen causing both healthcare-related and community-acquired infections with a substantial increase in morbidity and mortality. Though* S. aureus* can be isolated from various niches of human body, where it exists harmlessly as a commensal, it can also be an opportunistic pathogen in causing diverse array of infections ranging from skin and soft tissue lesions to lethal infections such as osteomyelitis, endocarditis, pneumonia, and septicaemia [3]. This commensal microflora readily colonizes the anterior nares and approximately 30% of healthy people carry this bacterium in their anterior nares [4]. As the nasal and extranasal colonization find chief prominence in the pathogenesis of invasive MRSA infections [5], studies on this pathogen from human throat (a least considered carriage site than the nares) are of dire need.

Besides,* S. aureus* is also widely known for its remarkable ability to infect and damage the indwelling medical prosthetics and other implants usually catheters through the fabrication of biofilm architectures [6, 7]. Another impressive characteristic feature of* S. aureus* in imposing such adverse clinical complications is its metabolic adaptability that facilitates the pathogen to colonize and persist in diverse environmental conditions. A wide range of virulent factors including extracellular toxins and surface structures in* S. aureus* are influential in the induction and persistence of infectivity within the host [8]. Although the potentials of biofilm assemblage of MRSA isolated from various infection sites of human and even from animals have been well demonstrated, studies on MRSA isolated from human throat are still inadequate. Therefore, the current study was proposed to characterize the biofilm-forming ability among clinical isolates of MRSA recovered from throat swabs pharyngitis patients.

The ability to attach, adhere, and synthesize biofilms has enhanced the virulence in MRSA. The mechanism of biofilm formation in* S. aureus* involves three major stages: initial attachment, maturation of biofilms, and dispersion of bacterial cells [9]. In* S. aureus* biofilm formation, the foremost and fundamental step is initial attachment, that is, adhesion which is being accomplished by the expression of different Microbial Surface Components Recognizing Adhesive Matrix Molecules (MSCRAMMs). These MSCRAMMs have high ability to interact with the host extracellular matrix proteins such as elastin binding protein (*ebpS*), laminin binding protein (*eno*), collagen-binding protein (*cna*), fibronectin-binding proteins A and B (*fnbA* and* fnbB*), fibrinogen binding protein (*fib*), and clumping factors A and B (*clfA* and* clfB*) [10]. Earlier studies on the molecular aspects of growth phase and subsequent establishment of biofilms have shown that* S. aureus* initially adhere to each other and then widen to structurally dynamic and intensely intricate biofilm architectures during the later phases of adherence. The biosynthesis of polysaccharide intercellular adhesin (PIA), a polysaccharide compiled from *β*-1, 6- linked N-acetyl-d-glucosamines (PNAG), is the hallmark element in the development of actual mature biofilms resulting in notorious multilayered clustering matrix of cells (second stage). PIA is mediated by the intercellular adhesin (*ica*) locus, which comprises four core genes, namely,* icaA*,* icaD*,* icaB*, and* icaC* and a regulatory gene (*icaR*) [6, 11]. The increase in the production of N-acetylglucosaminyl transferase and slime is facilitated by the coexpression of* icaA* and* icaD* genes [12]. While the genes* icaB* and* icaC* encode for extracellular membrane proteins, wherein* icaC* is whispered to have a role as receptor for polysaccharides and the function of* icaB* gene still remains uncover [13]. The accessory gene regulator (*agr*) locus, a well-characterized two-component regulatory system, plays a critical role in the upregulation and downregulation of protease and exotoxins, respectively [14], reflecting the final dispersal stage. In spite of deeper understanding on the biofilm-forming ability of* S. aureus*, it is still essential to extend the research on recently emerging MRSA strains (believed to be evolving from several clonal lineages of methicillin-susceptible* S. aureus* (MSSA) strains) as an attempt to address the complexity of their biofilm formation.

As a response to the above facts, the present study for the first time was focused on assessing the biofilm-forming properties among MRSA isolated from throat swabs of patients associated with pharyngitis through different phenotypic assays like slime synthesis,* in vitro* biofilm formation, and microbial adhesion to hydrocarbons (MATH). Furthermore, polymerase chain reaction (PCR) was performed to detect the adhesion (*icaA*/*icaD*/*icaB/icaC*), several adhesins (*fnbA*/*fnbB*,* clfA*, and* cna*), staphylococcal accessory regulator (*sarA*), and *α*-toxin (*hla*) genes. Finally, the* in vivo* adherence of the phenotypically and genotypically categorized MRSA isolates was assessed using a tropical nematode,* Caenorhabditis elegans,* as an animal model.

## 2. Materials and Methods

### 2.1. Bacterial Strains and Culture Conditions

A total of 63 MRSA isolates recovered from GAS associated pharyngitis patients were taken for evaluation of phenotypic and genotypic biofilm characteristics in the current study. The molecular identification and characterization of the MRSA isolates have already been done and reported by the same authors [15]. The MRSA isolates were grown and maintained on Tryptic soy agar/broth (TSA/TSB).

### 2.2. Phenotypic Assessment of Slime Synthesizing* S. aureus* Strains Using CRA

The qualitative slime production was assessed on the basis of the colour of* S. aureus* colonies developed on Congo red agar (CRA) plate according to the criteria described previously [16]. Briefly, MRSA clinical isolates were inoculated onto the CRA medium composed of TSB (30 g/L), sucrose (36 g/L), agar powder (18 g/L), and Congo red dye (0.8 g/L) and then cultured for 24 h at 37°C under aerobic conditions. The reference strains MRSA ATCC 33591 (slime producer) and* Staphylococcus epidermidis* ATCC 12228 (non-slime producer) were used as positive and negative controls, respectively.

The results regarding slime production were interpreted as follows: strains producing intensive black, black, and reddish black colonies with a rough, dry, and crystalline consistency were considered to be normal slime producers, whereas red and Bordeaux red with smooth colonies were classified as nonslime producers as reported elsewhere [17].

### 2.3. *In Vitro* Adherence Assay on Polystyrene Microtitre Plate (MtP)

*In vitro* biofilm formation was spectroscopically quantified by performing polystyrene microtitre plate (MtP) assay, as described previously with slight modifications [21]. Briefly, the test MRSA isolates were inoculated in 2 mL of TSB supplemented with 0.25% glucose and incubated overnight in shaking incubator (80 rpm, orbital shaker; Scigenics Biotech, Orbitek LEBT, India) at 37°C. The overnight culture of the test pathogens (1%) was then used to inoculate 24-well polystyrene MtPs containing 1 mL of fresh TSB supplemented with 0.25% glucose. The plates were incubated for 24 h at 37°C. After incubation, the plates were carefully washed thrice with sterile phosphate buffered saline (7 mM Na_2_HPO_4_, 3 mM NaH_2_PO_4_, and 130 mM NaCl at pH 7.4) to remove nonadherent cells and were air-dried in an inverted position before being stained. Adherent cells were stained with 1 mL of 0.4% crystal violet solution (w/v) for 2 min and the excess of dye was poured off. The wells were washed with sterile distilled water and then allowed to air-dry. Finally 1 mL of absolute ethanol was added into each well before being read spectroscopically. The optical density of the adherent biofilm was determined at OD_570 _nm, using a Multimode Microplate Reader (SpectraMax M3, USA) where the 1 mL of absolute ethanol served as blank. The strain* S. epidermidis* ATCC 12228 was used as the negative control. The adherence ability of tested isolates was classified into four categories based on the obtained OD: strongly adherent (OD_570_ ≥ 3.0), moderately adherent (OD_570_ ≤ 1.5–2.0), weakly adherent (OD_570_ ≤ 0.5–1.0), and nonadherent (OD_570_ < OD_570_ of negative control).

### 2.4. Confocal Laser Scanning Microscopy (CLSM)

In order to visualize the diverse biofilm architecture (on the basis of biofilm-forming potential through phenotypic and genotypic assays) of the four categorized test pathogens GSA-140, GSA-21, GSA-142, and GSA-54, Confocal Laser Scanning Microscopy (CLSM) (model: LSM 710) (Carl Zeiss, Germany) analysis was employed [22].

CLSM analysis was performed for the biofilms formed by the pathogens on glass pieces. The analysis was initiated by dispensing 1% inoculum of overnight cultures grown in TSB supplemented with 0.25% glucose into 24-well MtP containing 1 mL of fresh TSB + 0.25% glucose medium. Plates were statically incubated at 37°C for 24 h. After incubation, the glass pieces were gently washed with PBS and strained with 0.1% acridine orange for 5 min at room temperature in the dark. The stained glass pieces were gently washed thrice with PBS, air-dried, and observed under CLSM. Zen 2009 image software was used for analysis of biofilm images, which allowed for collection of* z*-stacks three-dimensional (3D) reconstruction. Images were acquired from random positions of biofilms formed on the glass slides. COMSTAT software (kind gift from Dr. Claus Sternberg, DTU Systems Biology, Technical University of Denmark) was used for further analysis of the obtained CLSM images (biofilm stack), in which three different parameters such as an average and maximum thickness (*μ*m) of the biofilms and the biovolume (*μ*m^3^), which is the volume of bacteria per *μ*m^2^ of glass surface used, were analysed [22].

### 2.5. MATH Assay

Cell surface hydrophobicity of the test pathogens was determined by using MATH (microbial adhesion to hydrocarbons) assay as an evaluation of their affinity towards the hydrophobic hydrocarbon (toluene) following the procedure described previously [23]. Briefly, 1 mL of test bacterial culture (OD_530 nm_ = 1.0) (Abs1) was placed into glass tubes along with 100 *μ*L of toluene. The mixtures were vigorously vortexed for 2 min and incubated for 10 min at room temperature to allow phase separation, and then the OD_530 nm_ of the aqueous phase was recorded (Abs2). The percentage of hydrophobicity was calculated according to the following formula: % hydrophobicity = [1 − (Abs2/Abs1)] × 100.

### 2.6. Detection of* icaA*,* icaD*,* icaB*,* icaC*,* fnbA*,* fnbB*,* clfA*,* cna*, and* hla* Genes

The chromosomal DNA of 63 MRSA isolates was extracted using the procedure described previously with minor modification [24] (omission of mutanolysin and hyaluronidase enzymes). The PCR assay for the detection of* icaA*,* icaD*,* icaB*,* icaC*,* sarA*,* fnbA*,* fnbB*,* clfA*,* cna*, and* hla* genes was performed using the primers (forward and reverse) and their respective standardized annealing temperatures as mentioned in Table 1. An aliquot of 2 *μ*L of DNA template (~10 ng) was added to 23 *μ*L of PCR mixture containing 1 × PCR buffer [10 mM Tris–HCl (pH 8.8), 50 mM KCl], 0.2 mM dNTPs, 1.5 mM MgCl_2_, 50 pM primer, and 1 U Taq polymerase (MBI Fermentas, Germany). Amplified PCR products were analyzed by agarose gel stained with ethidium bromide (0.5 *μ*g *μ*L^−1^) and visualized under ultraviolet transillumination and documented using Gel Doc XR apparatus (Biorad, USA).

### 2.7. *In Vivo* Adherence Assay Using* C. elegans*

A batch of three representative isolates was selected from each of the four categories (classified on the basis of phenotypic and genotypic characterization) for their* in vivo* adherence potential in* C. elegans*. The adherence assay was qualitatively examined by using CLSM as described earlier with slight modifications [25]. Briefly, twenty age-synchronized young adult hermaphrodite nematodes were transferred from a lawn of* E. coli* OP50 to the M9 buffer containing characterized MRSA isolates present in a sterile 24-well culture plate [20% inoculum (0.1 O.D of cells in 660 nm), i.e., 9 × 10^6^ cells m/L of LB medium] and incubated for 24 h at 20°C. After incubation, the nematodes were thoroughly washed and anesthetised by using 0.1 mM sodium azide to avoid expulsion of bacteria from nematodes intestine. Finally, the nematodes were stained with 0.1% acridine orange and visualized under CLSM.

### 2.8. Colony Forming Unit (CFU) Assay

To further ascertain the CLSM results and to quantify the adherence inside the* C. elegans*, a CFU assay was performed as described previously [25]. Briefly, a batch of ten nematodes were infected with each group of MRSA isolates (*n* = 3) for 24 h and washed thrice with M9 buffer to remove the surface bacteria. The washed nematodes were then transferred to the 1.5 mL microcentrifuge tube and the final volume was made up to 400 *μ*L with M9 buffer. Finally, 400 mg of silicon carbide particles (1.0 mm; Himedia, India) was added to each tube and vortexed at the maximum speed for 2 min. The resulting suspension was serially diluted and plated on Hicrome Aureus agar (Himedia, India) to determine the CFU.

## 3. Results

### 3.1. Phenotypic Characterization of* S. aureus* Slime Production on Congo Red Agar (CRA)

**Figure 1:**
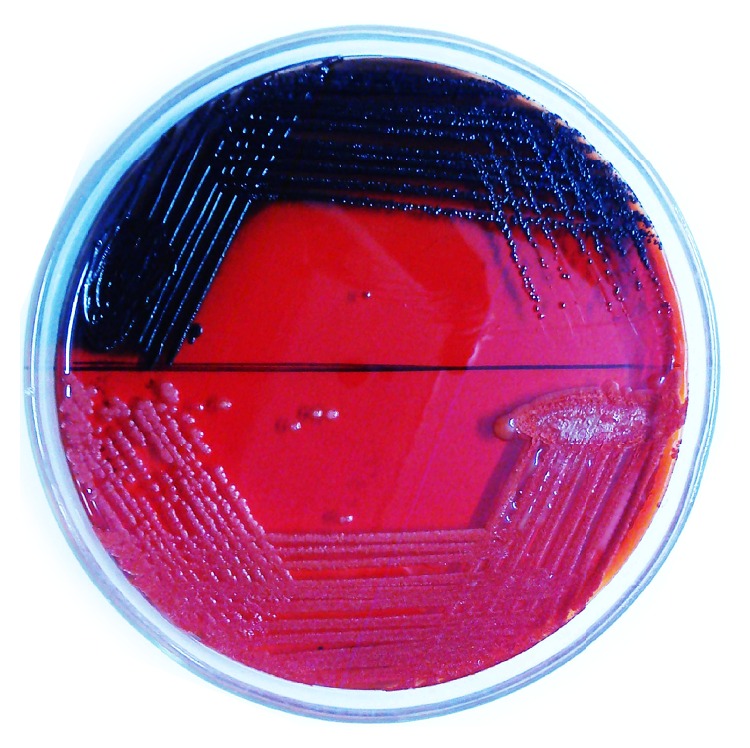
Colony morphologies of reference* Staphylococcus aureus* strains MRSA ATCC 33591 (positive control) and* S. epidermidis* ATCC 12228 (negative control) revealing strong black (upper sector) and pink (lower sector) coloured colonies on Congo red agar medium, respectively.

**Figure 2:**
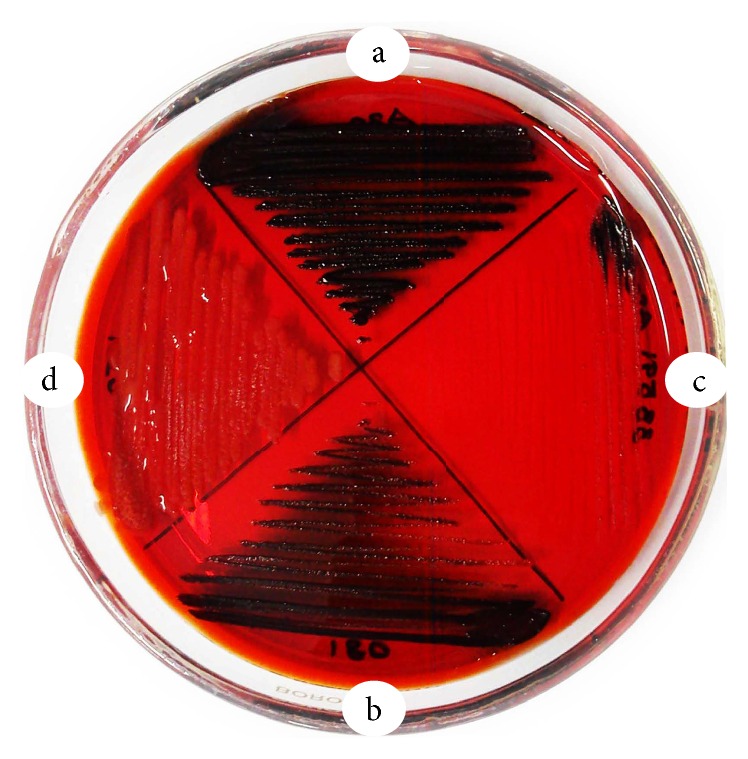
Congo red agar test showing four different slime producing patterns of clinical MRSA isolates: (a) slime positive bacteria with dark shiny black colonies (upper sector); (b) black colonies (bottom sector); (c) weak black colonies (right sector); and (d) slime negative bacteria showing pink coloured colonies (left sector).

The phenotypic determination of slime producing ability in Congo red agar of all the test isolates is shown in Table 2. As it is perceptibly evident from Figures 1 and 2 and Table 2, the different isolates of MRSA were unwaveringly found to be slime producers to varying degrees. Out of 63 MRSA isolates, 18 (28.6%), 23 (36.5%), 8 (12.7%), and 14 (22.2%) were determined to be strong black, black, reddish black, and Bordeaux red colour colony producers, respectively. The reference strains MRSA ATCC 33591 (positive control) and* S. epidermidis* ATCC 12228 (negative control) produced typical black and pink colonies, respectively, after 48 h incubation (Figure 1).

### 3.2. MATH Assay

The affinity of MRSA isolates towards toluene (nonpolar solvent) was unveiled by MATH assay and the results are summarized in Table 2. From the obtained results, it was found that the majority of the tested MRSA isolates (87.3%) exhibited a hydrophilic character, whereas eight MRSA isolates (12.7%) displayed a relative hydrophobic character.

### 3.3. *In Vitro* Adherence Assay on Polystyrene Microtitre Plate (MtP)

**Figure 3:**
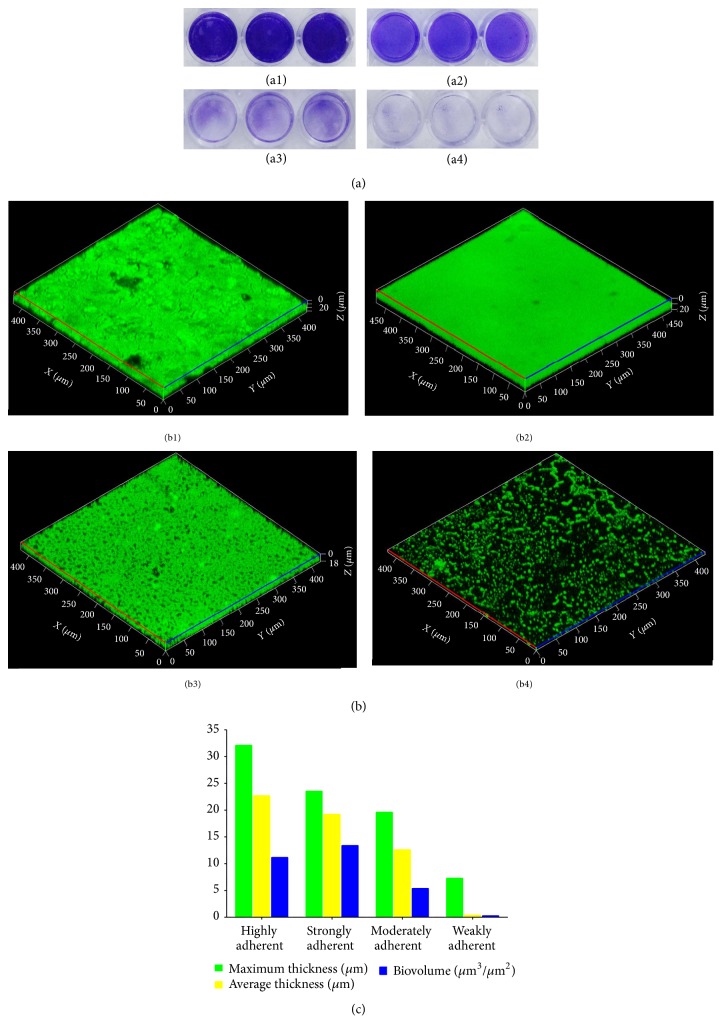
Total biofilm formation of different clinical MRSA isolates. (a) The bacterial cells were grown in 24-well Mtps containing TSB supplemented with 0.25% glucose. The cells that adhered to the plate surface after washing with phosphate buffer were visualized by crystal violet staining. The isolates were considered as highly adherent (a1), strongly adherent (a2), moderately adherent (a3), and nonadherent (a4) based upon their absorbance at 570 nm as measured by spectrophotometer. (b) Confocal laser scanning micrographs revealing variable degrees of biofilm production by clinical MRSA isolates on glass surface: (b1) highly adherent; (b2) strongly adherent; (b3) moderately adherent; and (b4) nonadherent isolates. (c) COMSTAT analysis of the obtained CLSM images.

The quantitative MtP method is the most extensively used gold standard technique for the detection of biofilm formation [26]. Table 2 and Figure 3(a) clearly show that all the MRSA isolates tested were found to be adherent at varying levels on 24-well polystyrene MtPs. Among 63 isolates studied, 21 (33.3%) isolates were highly adherent with OD_570_ values of >3, 5 isolates (7.9%) were strongly adherent with OD_570_ values of >2.0, 19 isolates (30.1%) were moderately adherent with OD_570_ values of >1–2.0, and 18 (28.6%) isolates were weakly adherent with OD_570_ values of <0.5–1. The MRSA ATCC 33591 strain was found to be strongly adherent with an OD_570_ value >2.0, while the* S. epidermidis* ATCC 12228 strain was negatively adherent (OD_570_ < 0.5).

### 3.4. Distribution of Adhesion and Biofilm Loci

**Figure 4:**
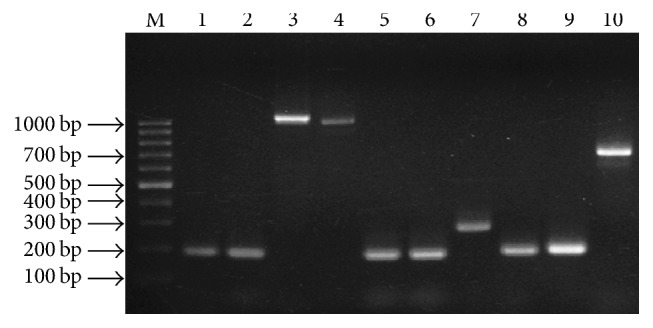
PCR amplification for the detection of genes responsible for biofilm formation in clinical MRSA isolates. Lane 1, 100 bp ladder (MBI Fermentas); lane 1–10, PCR amplicons of* icaA*,* icaD*,* icaB, icaC*,* fnbA*,* fnbB*,* clfA*,* cna*,* hla*, and* sarA* genes amplified from the clinical MRSA isolate GSA-32.

**Figure 5:**
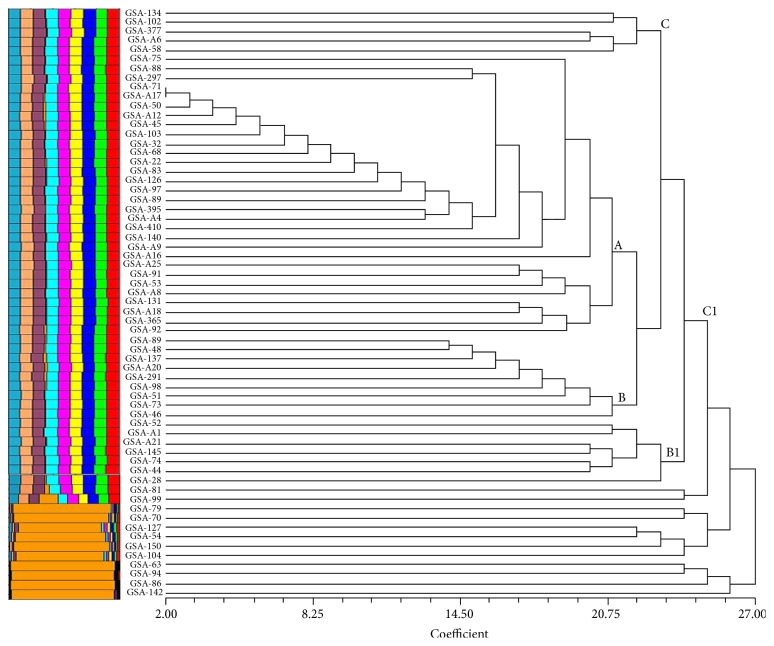
Dendrogram based on the amplification pattern of biofilm responsible genes, demonstrating the genotypic relatedness among the 63 MRSA isolates recovered from GAS pharyngitis patients. Scale represents the distance coefficient.

As the prime intention of the present study is the genotypic characterization of biofilm responsible genes, PCR assay was employed to detect* icaA*,* icaD*,* icaB*,* icaC*,* fnbA*,* fnbB*,* clfA*,* cna*,* hla*, and* sarA* genes among test MRSA strains. The distributions of these genes in 63 MRSA isolates are summarized in Table 2. As can be seen in Table 2, the majority of MRSA isolates [84.13% (*n* = 53)] were found to be positive for* icaADBC* genes. The prevalence of* sarA*,* fnbA*,* fnbB*,* clfA*,* cna*, and* hla* genes was unswervingly found to be 81, 84.1, 81, 58.7, 90.5, and 70%, respectively (Figure 4). Using the obtained biofilm responsible gene patterns of 63 MRSA isolates, a dendrogram was generated resulting in 5 clusters, namely, A, B, B1, C, and C1 (Figure 5). The data revealed that most of the strongly and moderately adherent isolates were under clusters B and B1 and around 95% of highly adherent isolates were harboured in cluster A, whereas clusters C and C1 showed the predominance of weak and few moderately adherent isolates.

### 3.5. *In Vivo* Adherence and Colonization of MRSA Isolates in* C. elegans*

**Figure 6:**
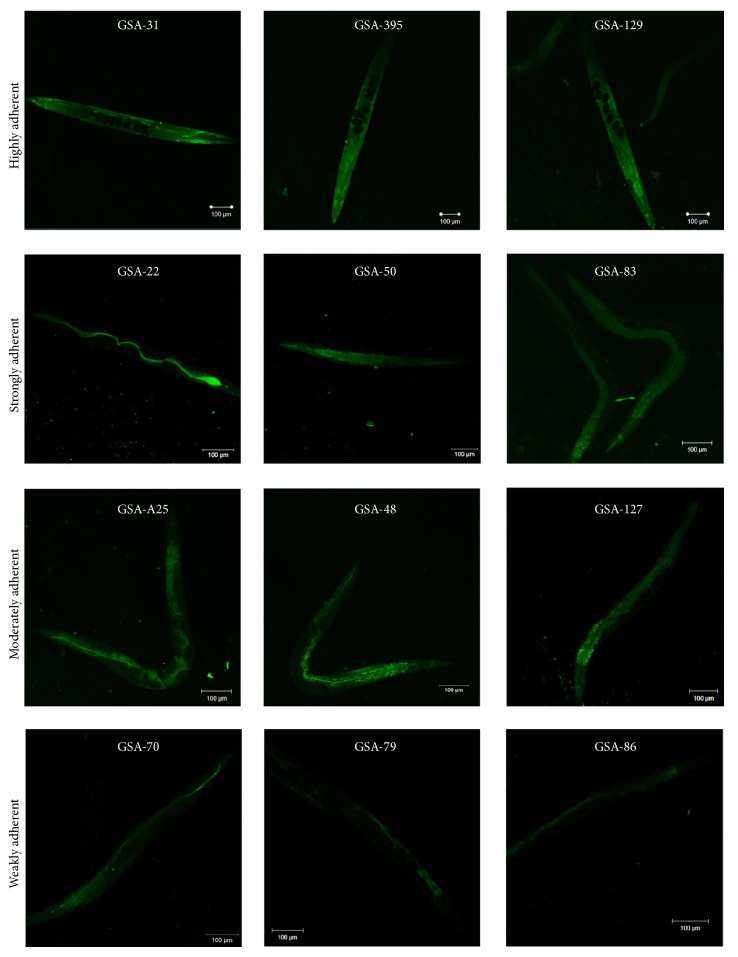
*In vivo* adherence and colonization of* C. elegans* infected with MRSA clinical isolates. Qualitative analysis of colonization in* C. elegans* infected with* S. aureus* clinical isolates using Confocal Laser Scanning Microscopy.

**Figure 7:**
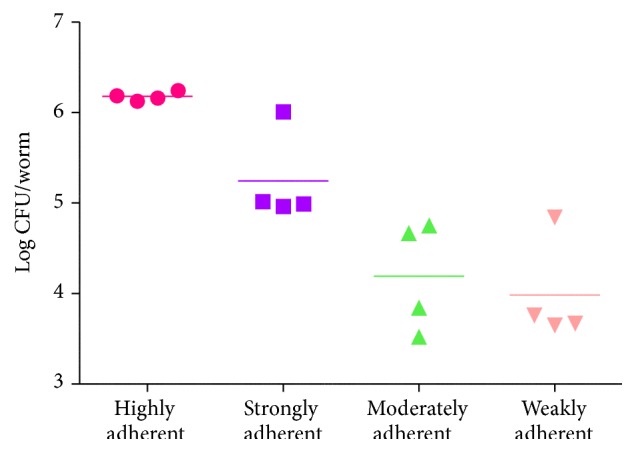
Presence of MRSA inside the* C. elegans*. Quantitative analysis of bacterial load inside the* C. elegans* exposed with MRSA clinical isolates.

In order to study the bioadherence property of four phenotypically and genotypically categorized MRSA isolates (highly, strongly, moderately, and weakly adherent isolates), an* in vivo* assay was performed using* C. elegans*. For examining the adherence potential of the MRSA isolates, the pathogen-exposed nematodes were examined by CLSM using Zen software. The fluorescence intensity found in the nematodes indicated the density of bacterial load inside the* C. elegans*. As anticipated, the highly and strongly adherent groups showed more intense fluorescence compared to the moderately and weakly adherent groups which showed moderate and very low fluorescence intensities, respectively (Figure 6). Furthermore, the level of CFU in pathogen-exposed nematodes was increased (152 ± 17.8  ×  10^4^) in highly adherent groups (*P* ≤ 0.05), modest (32 ± 4.6  ×  10^4^) in strongly adherent groups (*P* ≤ 0.05), and decreased in moderately (28 ± 2.6  ×  10^2^) and weakly adherent (21 ± 3  ×  10^2^) groups, respectively (Figure 7).

## 4. Discussion

Beyond being a commensal microflora,* S. aureus* primarily colonizes the anterior nares of human population. In addition, 30% (approximately) of healthy individuals are recognized as the carriers of this bacterium [4]. Though a few reports from the past have depicted that the human throat is less well studied site of carriage than the nares, apart from some isolations accounted, the scientific data obtained during 1940s have reported the throat colonization rate to be 4–63% [27]. Further persistent surveillance studies have reconfirmed the observation that MRSA in throat may be selectively colonized and escape from routine screening process in the infection control programs [28, 29]. Despite the fact that* S. aureus* was incredibly recurrent in causing varied range of human infections (aforementioned), the role of* S. aureus* in causing pharyngitis infection is also becoming noticeable but found less often when compared to the GAS pharyngitis infections [15, 30].

Though plethora of research findings have broadened our knowledge on the biofilm attributes of* S. aureus*, particularly MRSA emerging from various infection sites of human, it was necessarily important to widen our studies on the biofilm characterization of MRSA strains from new sites of infection as well. In our previous study, we demonstrated the possible role of MRSA on its own or in association with GAS in pharyngitis infection [15]. We extend the present study by performing the* in vitro* and* in vivo* biofilm characterization of the MRSA strains (*n* = 63), owing to the fact that the biofilm formation and adhesive ability are the prime virulence traits in* S. aureus*. The current study is the first of its kind to evaluate the biofilm-forming abilities among MRSA isolates recovered from new infection site, that is, throats of pharyngitis patients, which possibly would contribute towards the understanding of infection process. Researchers from the past have demonstrated the significance of MtP, CRA, and/or PCR techniques for the determination of critical virulence factors, particularly the ability of biofilm formation in* Staphylococcus* species [16, 31, 32].

Following the same paradigm, we also assessed 63 MRSA strains for their biofilm-forming capabilities employing three* in vitro* screening procedures (the MtP method, the CRA test, and the PCR technique). It has been well known that* S. aureus* can adhere and build biofilms on the medical implants and/or indwelling medical devices that can be attributed to a characteristic feature known as slime production [33]. This study utilized Congo red agar assay to determine the efficiency of test pathogens for their slime production, considering their high virulence and extreme potency in imposing severe postsurgical infections. Out of 63 MRSA strains tested, 49 (77.8%) were found to exhibit a positive phenotype for slime production by developing strong black or reddish black colonies on CRA plates. This result is in consonance with the previous reports by Kouidhi et al. [17], Arciola et al. [34], and Ammendolia et al. [35], wherein 50, 60.8, and 88.9% of* S. aureus* were found to be positive for slime production, respectively.

Cell surface hydrophobicity (CSH) plays a crucial role in the adherence of staphylococci to the host cells [17, 21]. Several reports from the recent past have reiterated this fact by observing that while there was a decrease in biofilm formation of* S. aureus*, similarly there was also a significant decrease in its cell surface charges like hydrophobicity during the treatment of any antibiofilm or sub-MICs of antibiotic agents [17, 22]. Here, we have determined the hydrophobic index of 63 MRSA isolates by performing MATH assay using toluene. The results summarized in Table 2 indicate that the surface affinity of* S. aureus* towards toluene was low signifying the hydrophilic nature of 87.3% (*n* = 54) of MRSA isolates subjected for this study. However, 12.7% (*n* = 8) of the isolates showed hydrophobicity and have also exhibited a strong biofilm formation on polystyrene MtPs, suggesting the possible interaction between the hydrophobic cells and substrate. The result of this assay is in agreement with the previous reports by Kouidhi et al. [17] and Hamadi et al. [36] portraying the hydrophilic nature of* S. aureus* surface.

Regardless of the actuality that several methods have been described so far to evaluate the accumulation and biofilm formation, MtP-based method was highly employed in most of the studies [37, 38]. The data of quantitative biofilm formation assay using MtPs showed 21 isolates as highly adherent (OD_570_ > 3), 5 isolates as strongly adherent (OD_570_ > 2.0 but <3), 19 isolates as low grade adherent (OD_570_ > 2), and remaining 18 as nonadherent (OD_570_ < 1). The result of this assay was validated by the confocal scanning micrographs (Figure 3(b)) followed by the COMSTAT analysis (Figure 3(c)) of the acquired images for single representative isolate from each of the four categories.

Further, the involvement of biofilms in clinical infections has received increasing interest due to the characterization of genes involved in biofilm formation [13]. Multitude of reports has demonstrated the significance of surface components in the biofilm formation of* S. aureus* such as the product of* icaADBC* operon, which encodes proteins for the synthesis of polysaccharide, poly-N-acetyl *β*-1-6-glucosamine (PNAG) [6, 39]. In addition, few extracellular proteins as well as cell-bound adhesins (also called MSCRAMMs) are considered essential for the pathogenicity of* S. aureus*. Consequently, the MRSA isolates were subjected to genotypic detection of* icaA*,* icaD*,* icaB,* and* icaC* genes and certain adhesin genes like* clfA*,* cna*,* fnbA,* and* fnbB* through PCR. The data of PCR analysis revealed that, except the 10 MRSA isolates, the remaining 53 MRSA isolates (84.13%) were found to harbour* icaADBC* genes. Our results were in total agreement with the recent studies stipulating that the percentage of* S. aureus* exhibiting* icaADBC* genotype was 100 [13]. Our findings were collinear with the observations by Atshan et al. [13] and Arciola et al. [12] as there was no difference in the prevalence of* icaADBC* genes in* S. aureus* with high and low virulence; however the only variation is found to be in the phenotypic characterization.

Conversely, adhesion to host cells requires genes like* fnb* (*A* and* B*),* clfA,* and* cna* that encode MSCRAMMs unlike the other factors involved in the adhesion to abiotic surfaces. Fibronectin-binding proteins (FnbA and FnbB) are large adhesins that may also function as invasins to modulate the adhesion and internalization of the organisms by different host cells. In addition, it has also been reported that fibronectin-binding facilitates the primary adherence and intercellular accumulation in biofilm assemblies [40]. In the present study, the distribution of* fnbA* and* fnbB* genes has been observed as 77.8% (*n* = 49) and 81% (*n* = 51), respectively, and around 73% (*n* = 46) of the MRSA isolates harboured both* fnbA* and* fnbB* genes (Table 2). A clinical study by Heilmann in 2011 [41] suggested that* S. aureus* strains associated with invasive disease were more likely to encode both* fnbA* and* fnbB* genes. Clumping factor (Clf) A and ClfB encoded by the genes* clfA* and* clfB* are the most important proteins for the binding of* S. aureus* to fibrinogen and fibrin; hence a mutant allele of* clfA* gene failed to clump and thus poorly adheres. In the present study, the* clfA* gene was present in 37 (58.7%) isolates, which was on a par with the previous report by Kohn et al. [42] suggesting that 89% of the test isolates are* clfA* positive. As aforementioned, collagen-binding proteins play an important role in the adhesion and pathogenesis of* S. aureus* [43]. In the current study, the presence of* cna* gene was found in 46 (73%) isolates, which was in agreement with other studies that reported the prevalence of* cna* gene as 46% [1] and 52% [44] in the isolates chosen for their study. However this is highly contrary with a report by Monecke et al. [45] suggesting that* cna* (collagen adhesin) was detected only in some clonal complexes. Staphylococcal alpha-hemolysin is one of the pore-forming toxins encoded by the gene* hla* which plays a major role in the biofilm formation and appears to be primarily required for cell-to-cell interactions. Therefore, a mutant allele of* hla* can initially aid in colonizing a substratum; however, it could not organize into multicellular macrocolonies. The PCR assay for the detection of* hla* gene revealed that 69.8% of (*n* = 44) MRSA strains were positive.

During the process of pathogenesis the chronological expression of several virulence determinants in* S. aureus* has been shown to be under the control of certain genetic loci, namely,* agr* (accessory gene regulator) and* sarA* (staphylococcal accessory regulator) [45]. In the midst,* sarA* is a chief global regulator that is essential for biofilm formation of MRSA and MSSA in both* in vitro* and* in vivo* conditions [46]. Since there has been a mounting evidence to suggest* sarA* as the positive regulator of PNAG-dependent biofilm formation in* S. aureus* [47, 48], in the present study the prevalence of* sarA* gene in MRSA isolates was assayed using PCR. The results revealed that the MRSA isolates harbouring the* icaADBC* genes were also positive for* sarA* gene, whereas the isolates with* icaADBC* negative genotypes were found negative for* sarA*, which is in corroboration with the findings from previous studies [47, 48]. The presence of* sarA* in 90.5% of MRSA strains from pharyngitis patients evidently implies the biofilm-associated pathogenic potential.

Furthermore, bearing in mind that* in vivo* adherence assay would be a better approach to comparatively assess the adhering ability of MRSA isolates with that of the phenotypic assays, three representative isolates from each of the four categories including highly, strongly, moderately, and weakly adherent groups were selected on the basis of their phenotypic and genotypic characteristics. The colonization by MRSA clinical isolates in* C. elegans* was localized using CLSM. The adherence of the pathogen in the host cell may possibly lead to the colonization of the pathogen in the host. As expected, the nematodes infected with highly adherent group showed an extensive intestinal colonization (Figure 6). On the other hand, the strongly adherent group exhibited more intense florescence compared to that of moderately and weakly adherent groups, which displayed very minimal fluorescence intensity. This was further authenticated with the results of CFU assay and therefore it is highly pertinent to state that the outcome of* in vivo* adherence assay clearly portrayed the factual frequency in the results obtained from* in vitro* adherence methods.

## 5. Conclusion

The data of the current study demonstrated the presence of* ica* genes, several adhesin genes, and the consequent phenotypic ability to form biofilm by most MRSA isolates. This biofilm-forming potential of MRSA isolates recovered from patients infected with pharyngitis in succession may facilitate and/or aggravate the infection, as such recalcitrant biofilms are 1000-fold more resistant to antibiotics and immune defence which may subsequently alleviate the pathogen to become multidrug resistant or may cause let-down in antibiotic therapy. In addition, the* in vivo* result suggests its good correlation with the findings of quantitative MtP method. Collectively, the outcome of the present study delineates, for the first time, the phenotypic (both* in vivo* and* in vitro*) as well as genotypic biofilm characterization of MRSA isolates recovered from GAS associated pharyngitis, which in turn ameliorates our perception and understanding of the pathogenesis and also its possible impact of causing throat infections.

## Figures and Tables

**Table 1:** Sequences of oligonucleotide primers used for PCR amplification of biofilm-associated genes.

Gene	Nucleotide sequence of primers (5′–3′).		Annealing temperature	Amplicon size (bp)	References
	Forward primer	Reverse primer			
*icaA* (intercellular adhesion gene)	ACACTTGCTGGCGCAGTCAA	TCTGGAACCAACATCCAACA	53°C	188	[17]
*icaD* (intercellular adhesion gene)	ATGGTCAAGCCCAGACAGAG	AGTATTTTCAATGTTTAAAGCA	53°C	198	[17]
*icaB* (intercellular adhesion gene)	CCCAACGCTAAAATCATCGC	ATTGGAGTTCGGAGTGACTGC	53°C	1080	[18]
*icaC* (intercellular adhesion gene)	CATGAAAATATGGAGGGTGG	TCAAACTGATTTCGCCCACCG	50°C	1000	[18]
*fnbA* (fibronectin-binding protein A)	ATCAGCAGATGTAGCGGAAG	TTTAGTACCGCTCGTTGTCC	55°C	198	[19]
*fnbB* (fibronectin-binding protein B)	AAGAAGCACCGAAAACTGTG	TCTCTGCAACTGCTGTAACG	55°C	198	[19]
*clfA* (clumping factor A)	ATTGGCGTGGCTTCAGTGCT	CGTTTCTTCCGTAGTTGCATTTG	55°C	292	[20]
*cna* (collagen-binding protein)	AAAGCGTTGCCTAGTGGAGA	AGTGCCTTCCCAAACCTTTT	55°C	192	[12]
*sarA* (staphylococcal accessory regulatory locus)	CCCAGAAATACAATCACTGTG	AGTGCCATTAGTGCAAAACC	53°C	720	[18]
*hla* (alpha toxin)	CAACTGATAAAAAAGTAGGCTGGAAAGTGAT	CTGGTGAAAACCCTGAAGATAATAGAG	50°C	200	[17]

**Table 2:** Relationships among the presence of *icaA*, *icaD*, *icaB, icaC*, *fnbA, fnbB, clfA, cna*, *hla,* and *sarA* genes, slime production, adherence capacity on MtPs, and hydrophobicity index in 63 different clinical MRSA associated with GAS pharyngitis infection.

Strain ID	Biofilm phenotype on CRA	Slime synthesis	*In vitro* adherence (MtP) assay		Hydrophobicity index ± SD	Presence of adhesion genes									
			Adherence OD_570_ nm ± SD	^*∗*^Adherence ability		*icaA*	*icaB*	*icaC*	*icaD*	*sarA*	*cna*	*clfA*	*fnbA*	*fnbB*	*hla*
GSA-83	Black	Producer	1.42 ± 0.213	++	26.3 ± 0.325	+	+	+	+	+	+	+	+	+	+

GSA-22	Black	Producer	1.79 ± 0.659	++	32.3 ± 0.336	+	+	+	+	+	+	+	+	+	+

GSA-32	Strong black	Producer	3.23 ± 0.986	+++	29.4 ± 0.962	+	+	+	+	+	+	+	+	+	+

GSA-A21	Black	Producer	1.53 ± 0.286	++	40.3 ± 0.560	+	+	+	+	+	+	−	+	−	−

GSA-127	Black	Producer	0.91 ± 0.632	+	32.9 ± 0.123	−	−	−	−	+	−	−	−	+	+

GSA-74	Strong black	Producer	3.21 ± 0.215	+++	41.3 ± 0.963	+	+	+	+	+	+	−	−	−	−

GSA-45	Strong black	Producer	3.09 ± 0.0236	+++	44.2 ± 0.023	+	+	+	+	+	+	+	+	+	+

GSA-150	Reddish black	Nonproducer	0.19 ± 1.023	−	15.3 ± 0.965	−	−	−	−	+	−	+	+	+	+

GSA-99	Reddish black	Nonproducer	1.32 ± 0.963	++	16.4 ± 0.189	+	+	+	+	−	+	−	−	+	+

GSA-103	Bordeaux red	Nonproducer	0.47 ± 0.451	−	21.6 ± 0.651	+	+	+	+	+	+	+	+	+	+

GSA-89	Strong black	Producer	3.41 ± 0.238	+++	29.3 ± 0.359	+	+	+	+	+	+	+	+	+	+

GSA-50	Black	Producer	3.06 ± 0.896	+++	31.2 ± 0.158	+	+	+	+	+	+	+	+	+	+

GSA-A8	Strong black	Producer	2.53 ± 1.639	++	27.9 ± 0.958	+	+	+	+	+	−	+	+	+	+

GSA-44	Strong black	Producer	3.86 ± 0.127	+++	24.6 ± 0.756	+	+	+	+	+	+	+	−	−	−

GSA-46	Bordeaux red	Nonproducer	0.39 ± 0.986	−	24.3 ± 0.286	+	+	+	+	+	+	−	+	+	+

GSA-48	Black	Producer	1.06 ± 1.028	+	20.9 ± 0.396	+	+	+	+	+	+	−	+	+	+

GSA-54	Bordeaux red	Nonproducer	0.49 ± 0.966	−	14.5 ± 0.362	−	−	−	−	+	−	+	−	+	+

GSA-395	Strong black	Producer	3.23 ± 0.523	+++	29.5 ± 0.396	+	+	+	+	+	+	+	+	+	+

GSA-68	Strong black	Producer	3.51 ± 0.889	+++	32.6 ± 0.325	+	+	+	+	+	+	+	+	+	+

GSA-94	Bordeaux red	Nonproducer	0.42 ± 0.365	−	18.6 ± 0.176	−	−	−	−	−	−	−	−	−	−

GSA-104	Black	Producer	1.48 ± 0.632	++	30.9 ± 0.963	−	−	−	−	−	+	+	−	+	+

GSA-140	Strong black	Producer	3.52 ± 0.023	+++	36.2 ± 0.990	+	+	+	+	+	+	−	+	−	−

GSA-145	Strong black	Producer	3.22 ± 0.965	+++	28.9 ± 1.230	+	+	+	+	+	+	−	+	−	−

GSA-142	Reddish black	Nonproducer	0.96 ± 1.036	+	30.6 ± 0.968	−	−	−	−	+	−	−	−	−	−

GSA-70	Bordeaux red	Nonproducer	0.23 ± 0.396	−	13.6 ± 0.869	−	−	−	−	−	−	−	+	+	+

GSA-126	Strong black	Producer	3.62 ± 0.325	+++	43.6 ± 0.310	+	+	+	+	+	+	+	+	+	+

GSA-A4	Black	Producer	2.57 ± 0.635	++	21.5 ± 0.256	+	+	+	+	+	+	+	+	+	+

GSA-92	Reddish black	Nonproducer	0.39 ± 0.961	−	12.9 ± 0.178	+	+	+	+	+	−	−	+	+	+

GSA-365	Black	Producer	1.98 ± 0.362	++	36.5 ± 0.986	+	+	+	+	+	−	+	+	+	−

GSA-A12	Black	Producer	1.59 ± 0.589	++	21.9 ± 0.936	+	+	+	+	+	+	+	+	+	+

GSA-A18	Bordeaux red	Nonproducer	1.09 ± 0.698	+	19.2 ± 0.129	+	+	+	+	+	−	−	+	+	−

GSA-297	Black	Producer	1.96 ± 0.129	++	29.9 ± 0.326	+	+	+	+	+	+	+	+	+	+

GSA-134	Reddish black	Nonproducer	3.12 ± 0.396	+++	13.6 ± 0.349	+	+	+	+	+	+	−	+	+	−

GSA-75	Black	Producer	1.79 ± 0.326	++	30.1 ± 0.559	+	+	+	+	+	+	+	+	+	+

GSA-88	Strong black	Nonproducer	3.09 ± 0.856	+++	24.3 ± 0.552	+	+	+	+	+	+	+	+	+	+

GSA-71	Black	Producer	3.85 ± 0.785	+++	22.3 ± 0.639	+	+	+	+	+	+	+	+	+	+

GSA-A25	Black	Producer	0.85 ± 0.759	+	26.8 ± 1.36	+	+	+	+	+	−	+	+	+	+

GSA-79	Bordeaux red	Nonproducer	0.21 ± 0.856	−	14.9 ± 0.759	−	−	−	−	−	+	−	+	+	−

GSA-52	Black	Producer	3.52 ± 0.996	+++	23.6 ± 0.529	+	+	+	+	+	+	+	−	−	+

GSA-91	Bordeaux red	Nonproducer	0.86 ± 1.236	+	15.9 ± 0.169	+	+	+	+	+	−	+	+	+	+

GSA-84	Strong black	Producer	3.11 ± 1.036	+++	40.1 ± 0.629	+	+	+	+	+	+	−	+	+	+

GSA-53	Bordeaux red	Nonproducer	0.36 ± 0.845	−	19.1 ± 0.785	+	+	+	+	+	−	+	+	+	+

GSA-137	Black	Producer	1.56 ± 0.965	++	22.6 ± 0.396	+	+	+	+	+	+	−	+	+	+

GSA-A20	Strong black	Producer	3.51 ± 0.515	+++	42 ± 0.968	+	+	+	+	+	+	−	+	+	+

GSA-291	Black	Producer	1.63 ± 0.689	++	26.9 ± 0.236	+	+	+	+	+	+	−	+	+	+

GSA-98	Black	Producer	1.79 ± 0.632	++	22.6 ± 0.756	+	+	+	+	+	+	−	+	+	+

GSA-73	Strong black	Producer	3.24 ± 0.325	+++	36.2 ± 0.688	+	+	+	+	+	+	−	+	+	+

GSA-A16	Black	Producer	2.06 ± 0.963	++	32.8 ± 0.895	+	+	+	+	−	+	+	+	+	+

GSA-131	Bordeaux red	Nonproducer	1.08 ± 0.896	+	16.3 ± 0.955	+	+	+	+	+	−	−	+	+	−

GSA-410	Strong black	Producer	3.69 ± 0.563	+++	36.8 ± 0.269	+	+	+	+	+	+	+	+	+	+

GSA-377	Bordeaux red	Nonproducer	0.27 ± 1.342	−	17.2 ± 0.745	+	+	+	+	+	+	+	+	+	−

GSA-A1	Black	Producer	3.04 ± 0.506	+++	21.1 ± 0.986	+	+	+	+	+	+	+	−	−	+

GSA-A6	Bordeaux red	Nonproducer	0.79 ± 0.966	+	13.9 ± 0.156	+	+	+	+	+	+	+	+	+	−

GSA-A9	Strong black	Nonproducer	2.89 ± 0.796	++	24.3 ± 0.969	+	+	+	+	+	+	+	+	+	+

GSA-A17	Black	Producer	1.85 ± 0.235	+++	19.9 ± 0.589	+	+	+	+	+	+	+	+	+	+

GSA-28	Bordeaux red	Nonproducer	1.25 ± 0.168	+	19.2 ± 0.129	+	+	+	+	+	+	+	−	+	−

GSA-51	Black	Producer	2.57 ± 0.234	++	21.3 ± 0.345	+	+	+	+	+	+	−	+	+	+

GSA-58	Reddish black	Nonproducer	0.55 ± 0.996	+	18.2 ± 0.569	+	+	+	+	+	+	+	+	+	−

GSA-63	Reddish black	Nonproducer	1.43 ± 0.351	++	11.9 ± 0.266	−	−	−	−	−	−	−	−	−	−

GSA-81	Reddish black	Nonproducer	0.98 ± 0.029	+	19.1 ± 0.192	+	+	+	+	−	−	−	−	−	+

GSA-86	Bordeaux red	Nonproducer	0.49 ± 0.259	−	16.2 ± 0.367	−	−	−	−	−	−	+	−	−	−

GSA-97	Strong black	Nonproducer	3.39 ± 0.125	+++	23.9 ± 0.121	+	+	+	+	+	+	+	+	+	+

GSA-102	Black	Producer	1.08 ± 0.985	+	24.5 ± 0.276	+	+	+	+	+	+	−	+	+	−

*∗*: Indicating the varied adhering ability of isolates on polystyrene surface, where +++ represents highly adherent (OD570 values of >3.0), ++ represents strongly adherent (OD570 values of >2.0), + represents moderately adherent (OD570 values of >1.0–2.0) and − represents weakly adherent (OD570 values of >0.5–1.0).
